# Serine 162, an Essential Residue for the Mitochondrial Localization, Stability and Anti-Apoptotic Function of Mcl-1

**DOI:** 10.1371/journal.pone.0045088

**Published:** 2012-09-14

**Authors:** Luke W. Thomas, Connie Lam, Richard E. Clark, Michael R. H. White, David G. Spiller, Robert J. Moots, Steven W. Edwards

**Affiliations:** 1 Institute of Integrative Biology, University of Liverpool, Liverpool, United Kingdom; 2 Institute of Translational Medicine, University of Liverpool, Liverpool, United Kingdom; 3 Institute of Aging and Chronic Disease, University of Liverpool, Liverpool, United Kingdom; University of Edinburgh, United Kingdom

## Abstract

Mcl-1 is an anti-apoptotic member of the Bcl-2 family that plays a key role in normal development, but also in pathologies such as cancer. It has some unusual properties compared to other anti-apoptotic members of the Bcl-2 family, and its expression and function are dynamically regulated by a variety of post-transcriptional and post-translational processes. Of note, Mcl-1 protein has a very short half life, and its stability and function may be regulated by reversible phosphorylation. There is also evidence to suggest that it may be localized to different subcellular compartments. The aim of this work was to determine whether residues within the PEST region of Mcl-1 that may undergo reversible phosphorylation, also regulate its subcellular distribution. We show that EGFP:Mcl-1 localizes mainly to the mitochondria of HeLa cells, with some additional cytoplasmic and nuclear localization. The mutations, S64A, S64E, S121A, S159A, T163A and T163E did not significantly affect the localization of Mcl-1. However, mutation of Ser162 to the phospho-null residue, Alanine resulted in an essentially nuclear localization, with some cytoplasmic but no mitochondrial localization. This mutant Mcl-1 protein, S162A, showed significantly decreased stability and it decreased the ability to protect against Bak-induced apoptosis. These data identify a new molecular determinant of Mcl-1 function, localization and stability that may be important for understanding the role of this protein in disease.

## Introduction

Mcl-1 is a crucial anti-apoptotic member of the Bcl-2 family with unique properties that distinguish it from other family members, such as Bcl-2 and Bcl-X_L_ [1]–[3]. It was first discovered as a gene induced early in the differentiation of ML-1 cells along the monocyte/macrophage pathway [4] and since this discovery, its importance and unique role in both normal physiology and pathology is becoming recognized. For example, Mcl-1 plays a key role in development, control of the cell cycle and resistance to apoptosis [5], [6] whereas defects in its expression underlie a number of pathologies, such as impaired development/maintenance of B- and T-lymphocytes [7] and enhanced apoptosis of differentiating monocytic U-937 [8]. Over-expression of Mcl-1 underpins malignancies that include multiple myeloma [9], hepatocellular carcinomas [10], colon carcinomas [11] and chronic myeloid leukaemia [12].

A key difference between Mcl-1 and other anti-apoptotic proteins of the Bcl-2 family is its relatively large size. The protein comprises 350 amino acids and residues 170–300 share structural/functional homology to both Bcl-2 and Bcl-X_L_, which comprise 239 and 233 residues, respectively [13]. In spite of its larger size, Mcl-1 possesses only 3 BH (Bcl-2 homology) domains, whereas both Bcl-2 and Bcl-X_L_ have 4. It also has a transmembrane domain at its C-terminus, deletion of which disrupts its ability to localize to sub-cellular membranes [14]. Despite this homology for part of the molecule, Mcl-1 differs from its anti-apoptotic counterparts in its binding affinities for pro-apoptotic proteins [15]. Mcl-1 has an extensive N-terminal region, not present in other family members, that confers many of its unique properties. This region contains 2 large PEST domains, several potential phosphorylation sites [16], [17], 2 caspase cleavage sites [18], and it is ubiquitinated by its own ubiquitin ligase (MULE) [19], which targets Mcl-1 for proteasomal degradation. Its half-life is ∼2–3 h in cultured and primary cell lines, which is extended in response to pro-survival agents, such as GM-CSF and bile acids [20], [21], and shortened in response to pro-apoptotic agents, such as sodium salicylate [22]. Alternatively, caspases regulate Mcl-1 turnover during accelerated apoptosis in response to high concentrations of TNFα, by activating caspases 3, 8, 9 and 10 [23]. Mcl-1 is thus a highly regulated anti-apoptotic protein, whose properties can be rapidly modulated via post-translational modifications, the majority of which occur in its unique N-terminal PEST region.

A number of the residues that regulate Mcl-1 turnover and stability have been mapped by mutagenesis, and these include key Ser and Thr residues that undergo reversible phosphorylation [3] and Lys residues that can become ubiquitinated [19]. A mitochondrial targeting motif, EELD has been identified [24], and in line with this observation, Mcl-1 localization is reported mainly at the mitochondria [14], [25]–[27] although some studies also show nuclear localization [28], [29]. This opens the possibility that Mcl-1 localization may be functionally important, although previous studies have not addressed this phenomenon.

The aims of the present study were to determine whether these key phospho-residues in the PEST domains of Mcl-1 may regulate, not only protein stability and turnover rates, but subcellular localization. This was achieved by site-directed mutagenesis and expression of GFP-linked wild type and mutant proteins, that were visualized by live cell confocal microscopy. We make the novel finding that mutagenesis of Ser162 to Ala, results in an essentially nuclear localization of Mcl-1. Moreover, this nuclear-localized protein is more unstable than the wild type protein and is less able to protect against apoptosis.

## Materials and Methods

### Cell Culture

All cell lines were obtained from ATCC : HeLa human cervical carcinoma cells were cultured in MEM (Gibco, UK) supplemented with 10% FBS, 100 U/ml penicillin, 100 U/ml streptomycin and 1% NEAA; HEK-293 human embryonic kidney cells, were cultured in DMEM (Gibco, UK) supplemented with 10% FBS, 100 U/ml penicillin, 100 U/ml streptomycin (Gibco, UK); MCF-7 cells were cultured as above, but were additionally supplemented with 5 µg/ml insulin and 1 µg/ml hydrocortisone.

### Plasmids

For experiments measuring the subcellular localization of Mcl-1, the following clones were generated. EGFP:Mcl-1 was generated by standard PCR techniques to introduce the desired restriction sites for ligation into pEGFP-C3 plasmid (Clontech, USA). Bak cDNA was generated to introduce the desired restriction sites for ligation into appropriately restriction digested pENTR-2A entry vector for use in the Gateway cloning system (Invitrogen, UK). Bak:EYFP was then generated by recombination of Bak:ENTR-2A with the appropriate EYFP destination vector. Mutant constructs were then generated by site-directed mutagenesis of Mcl-1:EGFP using the GeneTailor (Invitrogen, UK) whole plasmid PCR method. This generated Mcl-1 clones containing mutations in various phosphorylation sites that have previously been reported to alter the stability or function of Mcl-1. All mutants were verified by DNA sequencing.

For measurements of the turnover rate of wild type and mutant Mcl-1, the following protocol was adopted. Mcl-1 cDNA was first cloned into a pCMV-HA mammalian expression vector (Clontech USA) to generate a hemagglutinin epitope (HA)-tagged construct. HA-tagging of Mcl-1 allows distinction between exogenous expression of Mcl-1 from the endogenous protein by western blotting. HA:Mcl-1 was subsequently cloned into a pIRES2-EGFP vector (Clontech USA) using primers designed to anneal to hemagglutinin DNA. The generation of this HA-tagged pIRES-EGFP plasmid, which contains the internal ribosome entry site (IRES) of the encephalomyocarditis virus, permits the translation of two proteins, HA-Mcl-1 that has been cloned into the multiple cloning site and EGFP, from a single transcript.

### Transfection and Microscopy

For microscopy experiments, cells were plated on 35 mm glass-bottom microscopy dishes (Iwaki, Tokyo, Japan), at 75×10^3^ cells per dish in 2 ml growth medium and incubated at 37°C and 5% CO_2_. Cells were transfected with plasmids using Fugene 6 transfection reagent (Invitrogen) at a ratio of 2 µl Fugene 6 to 1 µg of DNA and cultured for 24 h prior to imaging. Confocal fluorescence microscopy was carried out on transfected cells using a Zeiss LSM510 Axiovert microscope with a 63× phase-contrast oil-immersion objective (numerical aperture = 1.4). Excitation of EGFP was performed using an Argon ion laser at 488 nm, and emitted light was reflected through a 505–550 nm bandpass filter from a 540 nm dichromatic mirror. EYFP fluorescence was excited using an Argon ion laser at 514 nm, passed through a 514 nm dichroic mirror and was collected through a 530 nm long-pass filter. MitoTracker Red (Molecular Probes, Invitrogen, UK) was added to cultures at 2 nM. Data capture and analysis was carried out with LSM510 version 3 software (Zeiss, Germany).

### Western Blotting

Protein stability of wild type or mutant Mcl-1 was assessed by incubating cells in the presence of cycloheximide (10 µg/ml) to inhibit *de novo* protein synthesis, and then measuring protein levels by western blotting. At each time point, cells were rapidly lysed using hot Laemmli buffer [5]. The HA antibody was utilized to analyze the half life of exogenously-expressed Mcl-1 and the mutants of interest, whilst the detection of EGFP was used to control for the variations in transfection efficiency between samples. Band intensities were measured using the AQM Advance 6 Kinetic Imaging program and data were expressed as a percentage of 0 h treatment following standardization against the EGFP control.

### Statistical Analysis

Data sets were analyzed using the paired Student’s t test.

## Results

### Subcellular Localization of Wild Type and Mutant Mcl-1

**Figure 1:**
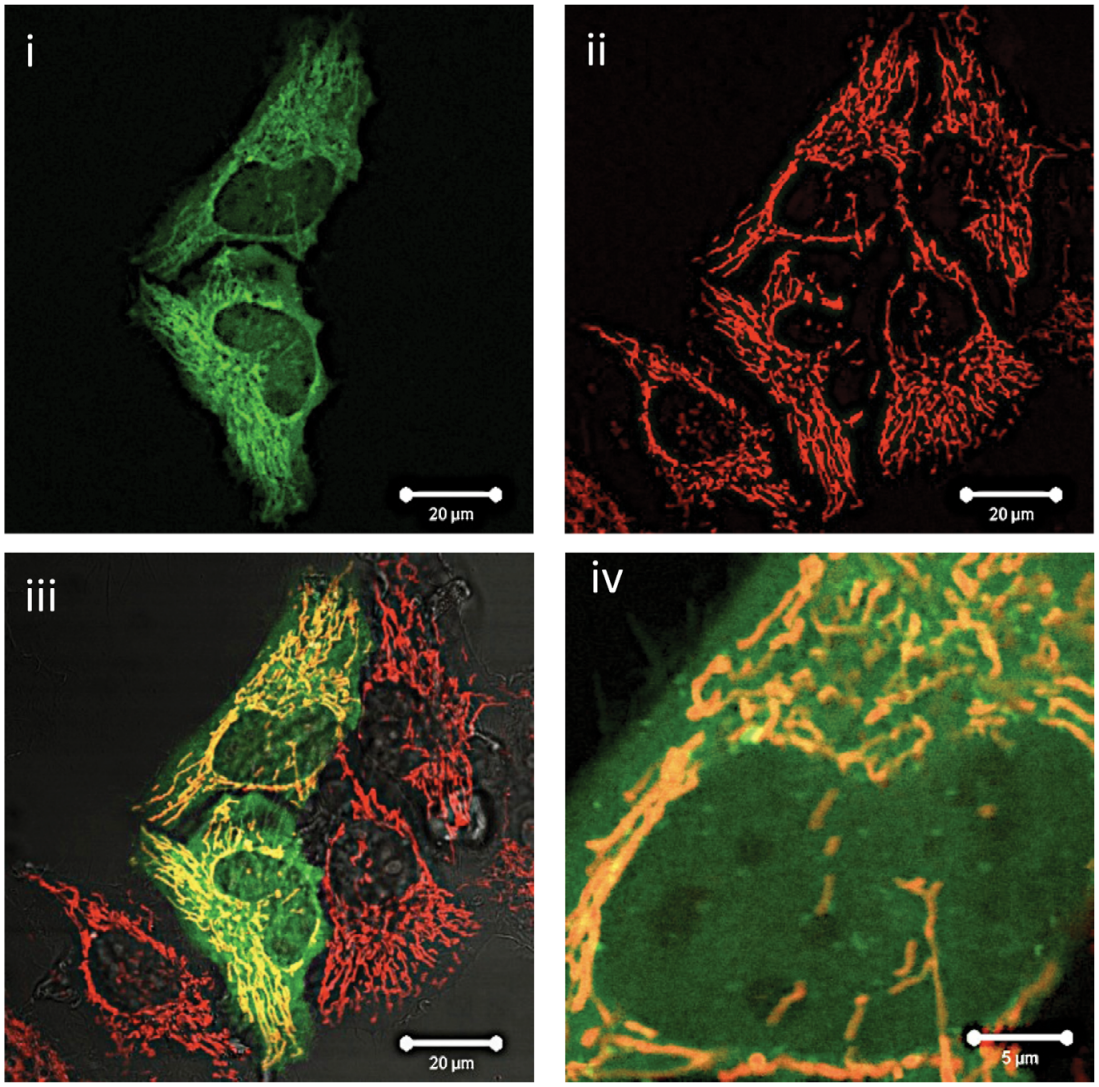
Subcellular localisation of wild type Mcl-1. Wild type EGFP-Mcl-1 was transfected and expressed in HeLa cells, co-stained with MitoTrackerRed and live cell confocal imaging was performed as described in Experimental. i, shows confocal image of EGFP localization, ii, MitoTrackerRed staining and iii, overlay of images i and ii. iv shows overlay images viewed at higher magnification. i–iii, bar marker represents 20 µm, while in iv, bar marker represents 5 µm. Images are representative of at least 20 separate transfection experiments.

Full length, wild type Mcl-1 localized primarily to the mitochondria of HeLa cells, confirmed by its co-localization with MitoTracker Red (Fig.1). All mitochondria co-stained for Mcl-1, but EGFP:Mcl-1 could also be detected in the cytoplasm and nucleus of the these cells, albeit at lower levels than found in mitochondria. This pattern of EGFP:Mcl-1 localization was seen in at least 25 separate transfection/imaging experiments and the non-mitochondrial cytoplasmic and nuclear staining was consistently observed, even when the transfected cells were expressing low levels of EGFP:Mcl-1. Fluorescence Recovery After Photobleaching (FRAP) experiments indicated that re-distribution of the low level of nuclear EGFP:Mcl-1 occurred rapidly after nuclear photobleaching (data not shown).

**Figure 2:**
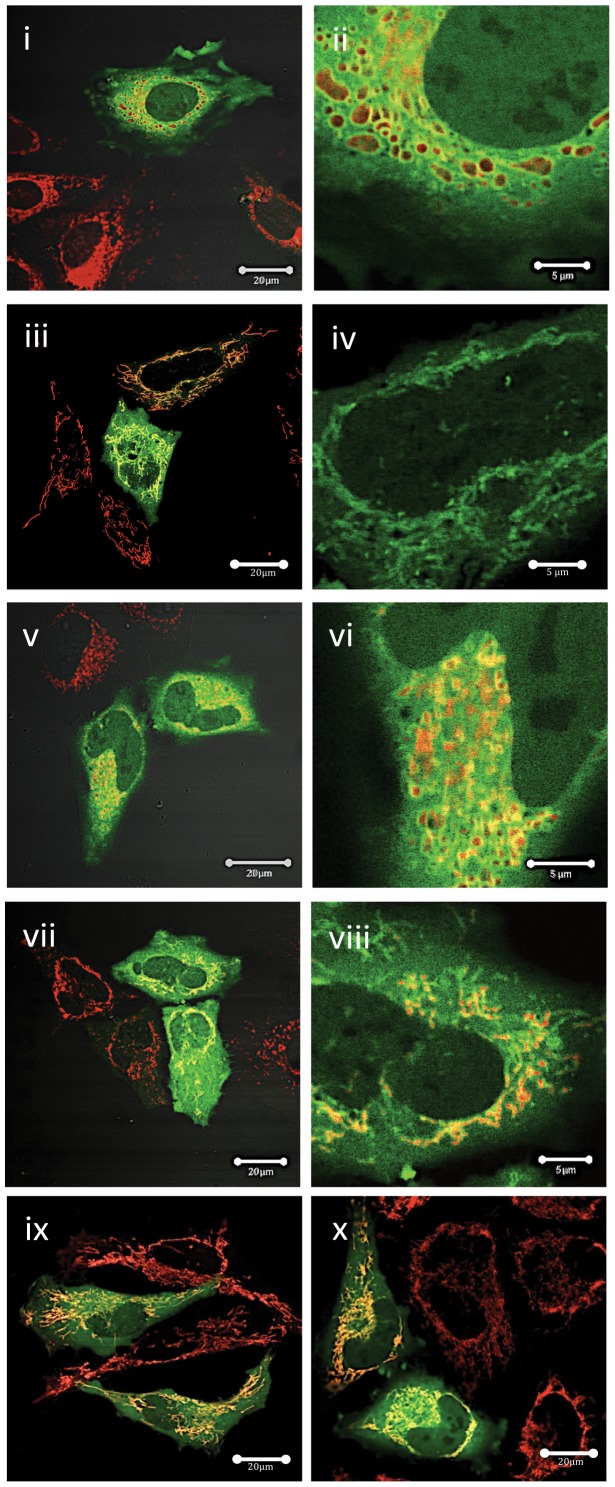
Subcellular localisation of mutant Mcl-1. Experiments were performed exactly as described in the legend to Figure 1. Images shown are all overlay images of EGFP-Mcl-1 and MitoTrackerRed. The following mutants are shown: i, ii, S64A; iii, iv, S64E; v, vi, S121A; vii, viii, S159A; ix, T163A, x; T163E. Bar marker in i, iii, v, vii, ix and x represents 20 µm, while in ii, iv, vi and viii it represents 5 µm. Images shown are representative of at least 10 separate transfection experiments with each mutant.

We then used site-directed mutagenesis to introduce mutations to phospho-residues that have been reported to alter the properties of Mcl-1, and then expressed these phospho-mutants in HeLa cells. Residue Ser64 of Mcl-1 is reported to be highly phosphorylated at the G2/M phase of the cell cycle by kinases such as CDKs 1 and 2, C-Jun terminal kinase and to protect cells from TRAIL-induced apoptosis [30]. The subcellular localization of Ser64A (Fig. 2i, ii) and the phospho-mimetic, Ser64E (Fig.2iii, iv), were very similar to those of the wild type protein, being localized to mitochondria, but with some additional cytoplasmic and nuclear localization. Serine 121 is reported to be phosphorylated by JNK in response to oxidative stress [31], [32], while Ser159 is phosphorylated by Glycogen Synthase Kinase (GSK) 3 [33]–[35]. Mutation of these residues to the phospho-null residue Ala, had no effect on the subcellular localization of Mcl-1 (Fig. 2 v–viii). Threonine 163 was the first phospho-residue of Mcl-1 to be shown to be phosphorylated by 12-O-tetradecanoyl phorbol-13-acetate (TPA) [17], [31], [34], [36]). Mutation of Thr163 to Ala did not affect its localization (Fig. 2ix) and similarly mutation of this residue to the phospho-mimetic, T163E did not affect its subcellular distribution (Fig. 2x).

**Figure 3:**
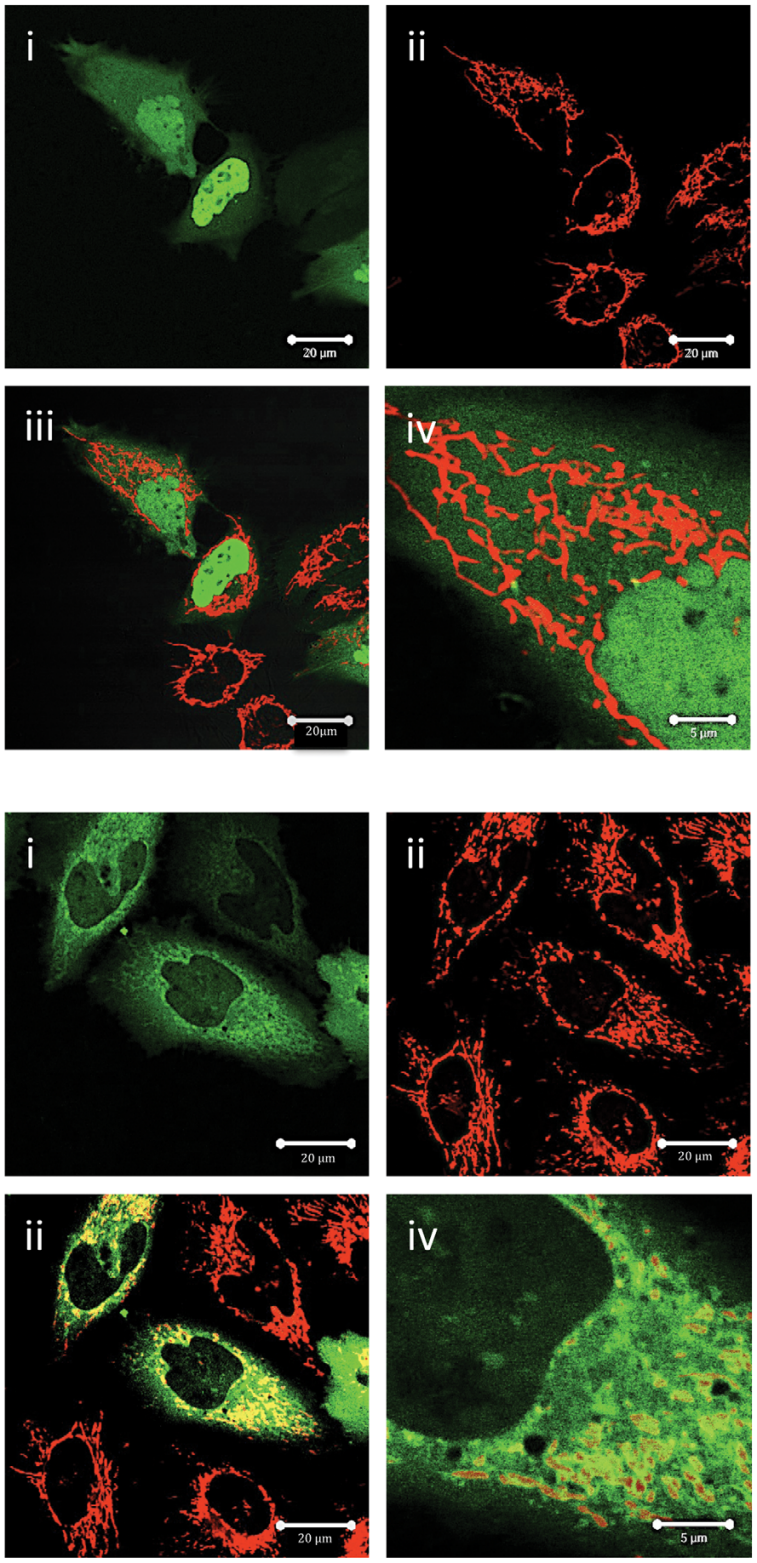
Subcellular localisation of S162 mutants. Experiments were performed exactly as described in the legend to Figure 1, except that HeLa cells were transfected with mutants of residue S162 of Mcl-1. Top panel shows localisation of S162A EGFP:Mcl-1 as follows: i, EGFP:Mcl-1; ii, MitotrackerRed; iii overlay and iv, enhanced magnification. Bar marker represents 20 µm in i-iii and 5 µm in iv. Bottom panel shows similar images but for S162E Mcl-1.

Adjacent to Thr163, and contained within a consensus MAPK site, is Serine 162. There are no previous reports of the properties of this residue on Mcl-1 function. When we mutated this residue to Ala (Ser162A), we noticed a remarkable shift in its subcellular localization (Fig.3 top panel). This Mcl-1 mutant was now localized primarily to the nucleus of HeLa cells, but was excluded from the nucleolus. Whilst there was clear, but low level cytoplasmic localization of this mutant protein, there was no detectable localization to the mitochondria (Fig. 3iv, top panel). This loss of mitochondrial localization and distribution to the nucleus was also observed when this mutant form of Mcl-1 was also transfected into other cells such as MCF-7 cells and Hek 293 cells (data not shown). The nuclear localization of this S162A mutant was confirmed in subcellular fractionation experiments. These experiments showed that endogenous Mcl-1 localized mostly to the non-nuclear fraction, whereas the S162A mutant (GFP:tagged) localized almost exclusively the nucleus (Figure S1). Furthermore, this subcellular fractionation experiment shows that nuclear localization of the S162A mutant does not affect the subcellular distribution of endogenous Mcl-1. When the phospho-mimetic S162E was generated and transfected into HeLa cells, it was found to localize similarly to the wild type protein, namely, mainly in the mitochondria, but with some additional cytoplasmic but minor nuclear staining (Fig. 3 bottom panel).

### Stability of Wild Type and Mutant Mcl-1 Protein

A number of the previously identified phospho-residues of Mcl-1 have been shown to affect its function, either by altering its ability to protect against apoptosis, or else by altering its rate of degradation [reviewed in 3]. The half life of the wild type of Mcl-1 protein has been measured in a number of cultured and primary cells, and protein stability is dynamically-regulated, so that this half life can be increased or decreased, depending upon the signals to which the cell is exposed [20], [22]. Various pathways of proteasomal- or non-proteasomal degradation can occur, and there is considerable evidence emerging to show that changes in the phosphorylation status of some key residues on the protein can affect its rate of turnover. Protein turnover rates can be measured by blocking *de novo* biosynthesis with cycloheximide, and then blotting for protein levels at various time points following the inhibitor block. This method is particularly suitable for measurement of the turnover rate of endogenously-expressed Mcl-1.

However, there are a number of potential technical problems associated with measuring the rate of turnover of exogenously-expressed proteins, because of varying levels of transfection of different batches of cells. For example, when we transiently-transfected HeLa cells with a plasmid allowing for the expression of wild type or mutant Mcl-1, variability of transfection rates between batches of cells, meant that no clear pattern of turnover rate could be determined when blots were corrected for expression of endogenously-expressed proteins such as actin or α-tubulin (data not shown). To overcome this problem, we developed a cloning strategy such that two exogenous proteins were expressed from a single plasmid. Mcl-1 (or mutants thereof) was first cloned into pCMV-HA, to generate a HA-tagged protein (to distinguish exogenous Mcl-1 from endogenous protein). Subsequently, HA:Mcl-1 cDNA was cloned into a pIRES-EGFP plasmid to express a secondary protein (EGFP), quantification of which served as a transfection control. Control experiments showed that EGFP expressed in HeLa cells was relatively stable, showing no loss of expression level following treatment of cells with cycloheximide for periods of up to 8 h (data not shown).

**Figure 4:**
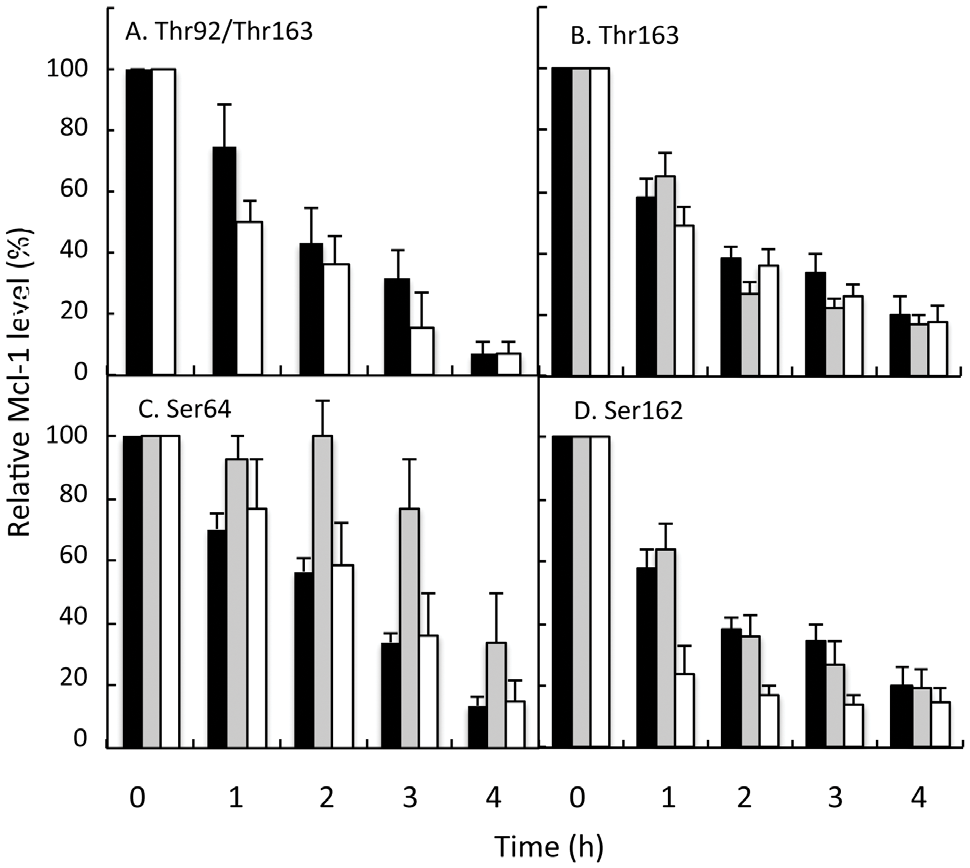
Stability of wild type and mutant Mcl-1 proteins. HeLa cells were transfected with a single plasmid allowing for the expression of HA:Mcl-1 (wild type or mutant) and EGFP. Suspensions were treated with 30 µg/ml cycloheximide, and at time intervals after the addition of this inhibitor of protein biosynthesis, samples were extracted in SDS-sample buffer prior to analysis of protein levels by western blotting. Expression of exogenous Mcl-1 was determined by probing with anti-HA antibodies, while EGFP-expression was determined with an anti-GFP antibody. Signals were quantified by densitometry and the level of expression of EGFP was used as a transfection control to normalize for Mcl-1 levels. In all panels () shows levels of wild type Mcl-1: in A the double T92A/T163A mutant is shown by (); in B, T163E shown by () and T163A by (); C, S64E by () and S64A by (); D, S162E by () and S162A by (). Number of replicates ranges from n = 3−8 in each panel.

Initial experiments were therefore conducted to determine the protein half life of exogenously-expressed Mcl-1 in HeLa cells and then to determine the effect of mutation of particular phospho-residues on protein stability. Figure 4 shows data validating this methodology with the half life of exogenously-expressed wild type Mcl-1 being 1.36 h ±0.2 h (determined by fitting the experimental data (n = 10) to first order decay kinetics). We then determined the stability of some Mcl-1 mutant proteins, focusing first on those residues that had been previously-reported to affect function or stability. First, we measured the half-life of a double mutation of T92A/T163A, as it has been reported that this double mutation results in decreased stability of the protein [36]. Figure 4 A confirms that Mcl-1 stability in this double mutant was decreased, thereby validating the methodology. Threonine 163 is a well-established phospho-residue of Mcl-1, that has been shown to be phosphorylated by agents such as TPA and ERK [17], [31], [34]. While its importance in stabilizing Mcl-1 when phosphorylated by TPA has been inferred, this has not been directly demonstrated experimentally. The data in Figure 4B show that mutation of Thr163 to either A or the phospho-mimetic, E resulted in only small changes in Mcl-1 stability, but these did not reach statistical significance. Serine 64 has been identified as a target of CDKs 1 and 2, C-Jun N-terminal kinase (JNK) and to be strongly phosphorylated in the G2/M phase of the cell cycle [30]. The data in Figure 4C show that whereas the mutant S64A had similar stability to the wild type protein, the phospho-mimetic, S64E demonstrated considerably enhanced stability.

The effects of mutation of Ser162 on the turnover rate of Mcl-1 were then determined. Mutation of this residue to the phospho-mimetic, Ser162E, did not alter the rate of degradation, compared to the rate of turnover of the wild type protein. However, the turnover rate of the phospho-mutant S162A was significantly enhanced compared to wild type protein (Fig. 4D). After 1 h following cycloheximide addition, protein levels had decreased by about 80%, indicating a half life of approx. 30 min. Hence, these data suggest that this residue is responsible for regulating both the subcellular location and the turnover rate of Mcl-1.

### Role of Ser162 in Protection Against Apoptosis

**Figure 5:**
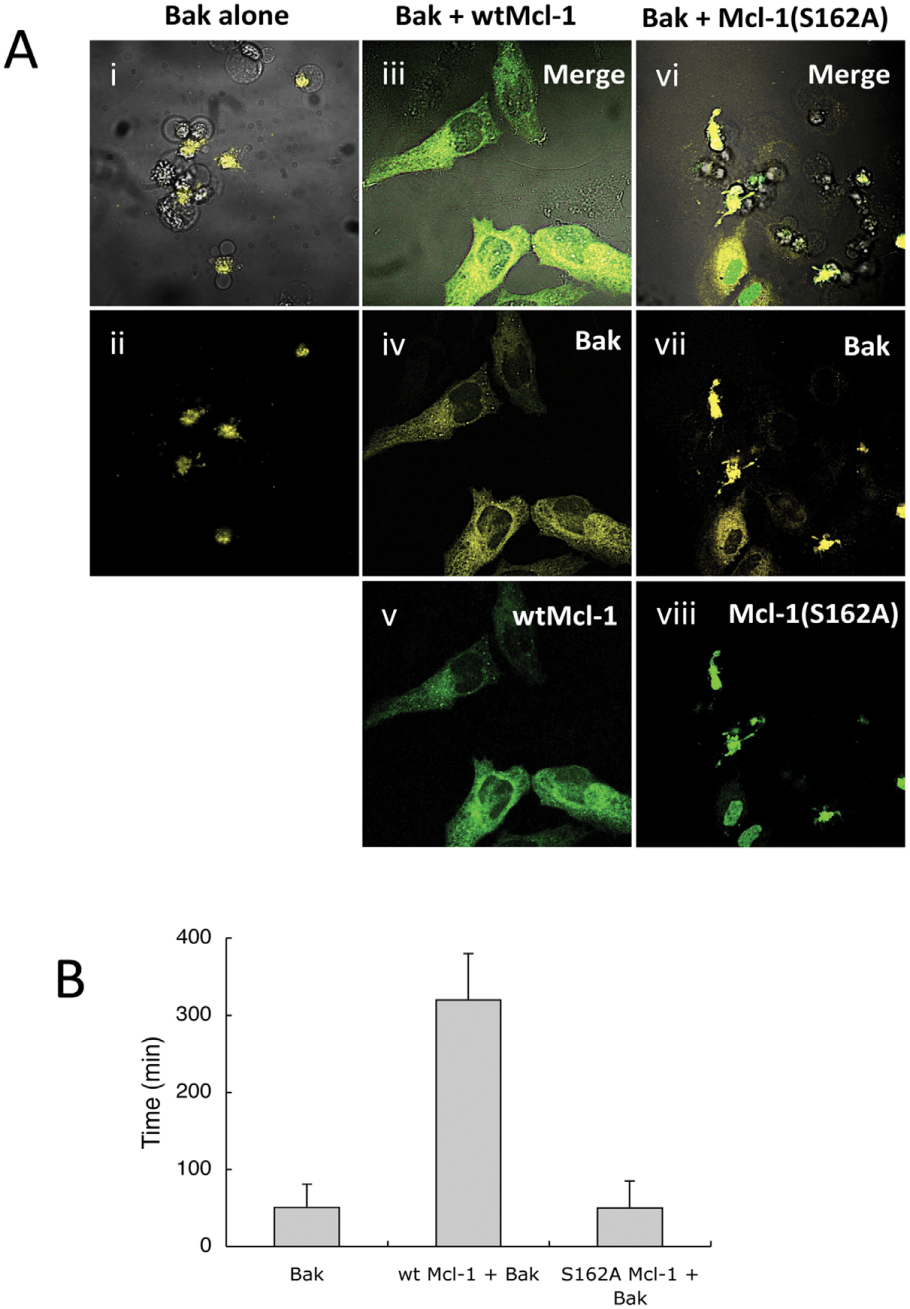
Protection from apoptosis wild type and S162A Mcl-1. In A, HeLa cells were transfected to express YFP:Bak, either alone (i, ii) or with wild type (iii-v) or S162A (vi-viii) Mcl-1. Images were taken from a time lapse series experiment for 24 h post transfection, as follows: (i) YFP:Bak fluorescence plus merged bright field; (ii), YFP:Bak (showing apoptosis in cells expressing the pro-apoptotic protein; (iii), merged wild type EGFP:Mcl-1 and YFP:Bak; (iv), YFP:Bak; (v) wild type EGFP:Mcl-1; (vi) merged bright field and fluorescence images of YFP:Bak and S162A EGFP:Mcl-1; (vii) YFP:Bak and (viii) S162A EGFP:Mcl-1. B shows quantitative data of survival times of cells in culture following transfection with YFP:Bak, YFP:Bak plus wild type EGFP:Mcl-1 and YFP:Bak plus S162A EGFP:Mcl-1. n = 54.

We next designed experiments to determine whether the nuclear localization and high turnover rate of mutant S162A Mcl-1 also impaired its ability to protect against apoptosis. For these experiments, we transfected HeLa cells with YFP-Bak so that we could assess apoptosis and protein expression/localization by confocal microscopy. When HeLa cells were transfected with YFP-Bak alone, time-lapse experiments revealed that apoptosis was initiated within minutes of the cells beginning to express this apoptotic protein (data not shown). All cells expressing YFP-Bak showed signs of apoptosis (Fig. 5A). When YFP-Bak was co-expressed with wild type EGFP-Mcl-1, the cells showed normal survival, suggesting that the pro-apoptotic effects of exogenous Bak expression were counteracted by the co-expression of exogenous wild type Mcl-1 (Fig. 5B). However, when YFP-Bak was co-expressed with S162A EGFP-Mcl-1, then two observations were made. Firstly, the mutant Mcl-1 protein appeared to be retained within the nucleus and secondly, the cells were not protected against apoptosis (Fig. 5C, D).

## Discussion

In this report, we make the novel discovery that Ser162 is a key regulator of the localization, turnover rate and function of Mcl-1, and an important regulator of apoptosis. It is now established that Mcl-1 has many unique properties compared to other anti-apoptotic members of the Bcl-2 family, and that many of the molecular motifs regulating these properties reside within the large N-terminal domain of the protein that contains PEST domains. This region contains proteolytic cleavage sites (for caspases and other proteases), multiple phospho-residues (that control turnover rate and function) and Lys residues (that become ubiquitinated prior to proteasomal targeting and degradation). In addition to these post-translation modifications affecting function, Mcl-1 expression is also regulated by alternative splicing (leading to the expression of proteins with either pro-or anti-apoptotic properties), changes in transcript stability and also translational control, regulated by the binding of mir-29b and CUGBP2 to the 3′-UTR of the transcript [37], [38]. All of these regulatory processes suggest that Mcl-1 plays a unique and central role in the dynamic regulation of cell death and survival in response to external signals.

Mcl-1 is proposed to interact with BH3 proteins such as Bak on the mitochondrial membrane preventing such molecules from dimerizing to form pro-apoptotic pores and subsequent cytochrome c release. Our data (e.g. Fig.1), and that of other groups, indicate a strong mitochondrial localization of wild type Mcl-1, in accord with this anti-apoptotic function. However, Mcl-1 is also localized to the cytoplasm and to a lesser extent, the nucleus of many cells. We hypothesized that localization to these other non-mitochondrial sites might be important for its function in cell survival. A cytosolic localization of Mcl-1, for example, might be required to sequester cytosolic Bax or similar proteins, while a nuclear localization has been proposed to be required for interactions with CDK-1 and PCNA, suggesting a role for Mcl-1 in cell cycle regulation or DNA repair [29], [39]. While the molecular motifs regulating the mitochondrial localization of Mcl-1 are becoming defined [24], those regulating cytoplasmic or nuclear localization remain unclear.

We show here that the subcellular distribution of the null phospho-mutant, S162A is completely different to that of the wild type protein, being localized primarily to the nucleus rather than the mitochondria (Fig. 1, 3). However, a cytoplasmic localization of S162A was still observed. These data suggest that changes in the phosphorylation status of Mcl-1 on Ser162 may be the molecular “switch” that changes its localization from an essentially mitochondrial location to a mainly nuclear distribution. We also show that this change in sub-cellular distribution is associated with changes in the function of this protein as the turnover rate and ability to protect against Bak-induced apoptosis is altered in the S162A mutant. These observations point to a key role for this residue in regulating cell fate via reversible phosphorylation. We propose that nuclear-localized Mcl-1, not phosphorylated on Ser162, is unable to protect against pro-apoptotic events that occur on the mitochondria. The corollary of this observation is that phosphorylation of Ser162 is required for a mitochondrial localization and hence functional of role of Mcl-1 in apoptosis protection.

As part of our studies to determine the half-life of the S162A mutant protein compared to that of the wild type protein, we designed a new cloning/expression protocol that enabled us to correct hybridization signals in western blots for variations in transfection efficiency between different batches of cells. As our mutagenesis approach necessitated the screening of many site-directed mutants for Mcl-1, it was not feasible to generate stably transfected cell lines for each of the mutants. Instead, our screening strategy utilized transient transfections, but we encountered significant difficulties in “normalizing” western blot signals between different batches of cells of varying transfection efficiencies. We therefore developed a system whereby two proteins, HA-tagged Mcl-1 (or mutants thereof) and EGFP were expressed from a single transcript using an IRES-containing bicistronic vector. This enabled us to probe for levels of exogenous Mcl-1 (without interference from endogenous Mcl-1) using an anti-HA antibody, and to correct for transfection efficiency using an anti-EGFP antibody.

Our first experiments showed that the rate of turnover of wild type Mcl-1 is 1.65 h±0.2 h, in line with many other reports that this protein has a rapid rate of turnover in a variety of cell lines. It was then necessary to determine if this assay had the sensitivity to detect changes in degradation rates that resulted from mutagenesis of specific residues. We therefore measured the turnover rates of some Mcl-1 mutants that had been characterized in other experimental systems. First, we showed that the double mutant T92A/T163A had an increased rate of protein turnover, confirming a previous report [36]. However, we did not measure any change in turnover rate of either a T163A or T163E mutant, compared to that of wild type Mcl-1. It has previously been reported that TPA treatment of BL41-3 cells enhanced Mcl-1 stability and phosphorylates Thr163 [17] and TPA-induced Mcl-1 phosphorylation was decreased in a T163A mutant. It was therefore inferred (but not experimentally tested) that phosphorylation of Thr163 confers enhanced Mcl-1 stability. A number of other reports have confirmed Thr163 phosphorylation and determined its function, but usually in conjunction with a second mutation, such as Ser121, Ser155 or Ser159 [31], [34], [36]. Apart from the differences in cell lines used, our experiments also differ in that we measured the effect of these mutations on the endogenous, rather that TPA-regulated, turnover of wild type or mutant Mcl-1.

Another finding from our study was the significantly-enhanced stability of the S64E Mcl-1 mutant. Previous studies on this residue have suggested its ability to undergo reversible phosphorylation, and showed that a S64E mutant had enhanced stability in KMCH cells (with endogenous Mcl-1 knocked down by shRNA treatment). However, this change in stability was reported by the authors to be non-significant [30]. In HeLa cells, we show that after a 3 h incubation with the protein synthesis inhibitor, cycloheximide, around 80% of the protein was still detected, whereas by this time, only around 30% of the wild type protein remained. Thus, we found that the most dramatic changes in the stability of Mcl-1 protein were regulated by Ser162 (with S162A being the most unstable form of the protein) and Ser64 (with S64E being the most stable form). Differential phosphorylation on these two residues by differential kinase/phosphatase activity, would therefore lead to dramatic changes in protein stable stability. This could have major effects on cell survival and apoptosis. It is now necessary to identify the kinase/phosphatase systems responsible for phosphorylation/dephosphorylation of Serine 162 and to determine the patho-physiological stimuli that may control these activities. Such information could be important for designing new ways to induce apoptosis in cancer cells in which abnormal Mcl-1 expression underpins pathology.

## Supporting Information

Figure S1 **Non-transfected (control) and EGFP:Mcl-1 (S162A mutant) HeLa cells were fractionated into cytoplasmic and nuclear fractions as using Thermo Scientific NE-PER Nuclear and Cytoplasmic Extraction Reagents, as per the manufacturer’s instructions (** http://www.piercenet.co **).** Following SDS-PAGE and transfer to PVDF membranes as described in [23], cytoplasmic (C) and nuclear (N) extracts of control (NT) and EGDP:Mcl-1 (S162A) transfected cells were blotted with the following antibodies: Mcl-1 (which recognizes the 40-kDa endogenous protein and the 60-kDa exogenous (EGFP-tagged protein); EGFP (which recognizes only exogenous Mcl-1); α-tubulin (a 50-kDa cytoplasmic protein); lamin B (a 67-kDa nuclear protein).
(TIF)
