# Supplementary figures and images for: Serine 162, an Essential Residue for the Mitochondrial Localization, Stability and Anti-Apoptotic Function of Mcl-1
Source: PLoS One. 2012 Sep 14;7(9):e45088. doi: 10.1371/journal.pone.0045088 (PMC3443205; doi:10.1371/journal.pone.0045088)

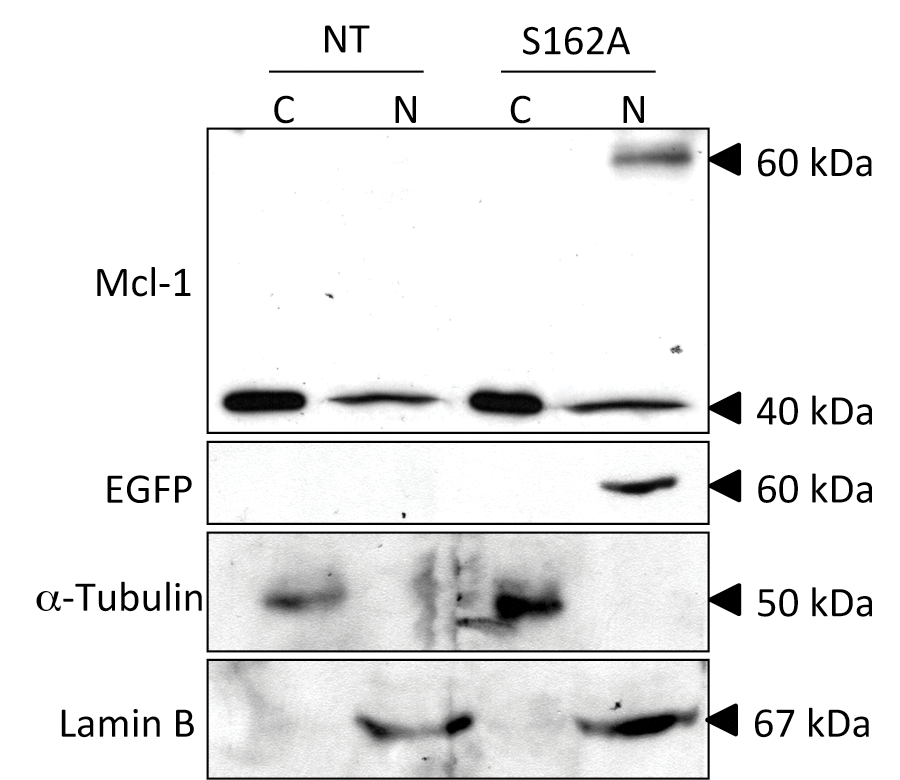

Supplement: Figure S1 — Non-transfected (control) and EGFP:Mcl-1 (S162A mutant) HeLa cells were fractionated into cytoplasmic and nuclear fractions as using Thermo Scientific NE-PER Nuclear and Cytoplasmic Extraction Reagents, as per the manufacturer’s instructions ( http://www.piercenet.co ). Following SDS-PAGE and transfer to PVDF membranes as described in [23], cytoplasmic (C) and nuclear (N) extracts of control (NT) and EGDP:Mcl-1 (S162A) transfected cells were blotted with the following antibodies: Mcl-1 (which recognizes the 40-kDa endogenous protein and the 60-kDa exogenous (EGFP-tagged protein); EGFP (which recognizes only exogenous Mcl-1); α-tubulin (a 50-kDa cytoplasmic protein); lamin B (a 67-kDa nuclear protein). (TIF) [file pone.0045088.s001.tif]
